# Recent Progress on Systems and Synthetic Biology of Diatoms for Improving Algal Productivity

**DOI:** 10.3389/fbioe.2022.908804

**Published:** 2022-05-13

**Authors:** Jiwei Chen, Yifan Huang, Yuexuan Shu, Xiaoyue Hu, Di Wu, Hangjin Jiang, Kui Wang, Weihua Liu, Weiqi Fu

**Affiliations:** ^1^ Department of Marine Science, Ocean College, Zhejiang University, Hangzhou, China; ^2^ Center for Data Science, Zhejiang University, Hangzhou, China; ^3^ School of Mathematical Sciences, Zhejiang University, Hangzhou, China; ^4^ Center for Systems Biology and Faculty of Industrial Engineering, Mechanical Engineering and Computer Science, School of Engineering and Natural Sciences, University of Iceland, Reykjavik, Iceland

**Keywords:** microalgae, diatoms, synthetic biology, systems biology, photosynthesis, biomass productivity

## Abstract

Microalgae have drawn much attention for their potential applications as a sustainable source for developing bioactive compounds, functional foods, feeds, and biofuels. Diatoms, as one major group of microalgae with high yields and strong adaptability to the environment, have shown advantages in developing photosynthetic cell factories to produce value-added compounds, including heterologous bioactive products. However, the commercialization of diatoms has encountered several obstacles that limit the potential mass production, such as the limitation of algal productivity and low photosynthetic efficiency. In recent years, systems and synthetic biology have dramatically improved the efficiency of diatom cell factories. In this review, we discussed first the genome sequencing and genome-scale metabolic models (GEMs) of diatoms. Then, approaches to optimizing photosynthetic efficiency are introduced with a focus on the enhancement of biomass productivity in diatoms. We also reviewed genome engineering technologies, including CRISPR (clustered regularly interspaced short palindromic repeats) gene-editing to produce bioactive compounds in diatoms. Finally, we summarized the recent progress on the diatom cell factory for producing heterologous compounds through genome engineering to introduce foreign genes into host diatoms. This review also pinpointed the bottlenecks in algal engineering development and provided critical insights into the future direction of algal production.

## 1 Introduction

Diatoms are natural cell factories that synthesize various value-added compounds, such as polyunsaturated fatty acids, pigments, terpenes, and sterols (Yang et al., 2020). Recent years have witnessed tremendous progress in genome sequencing and editing of diatoms. The genome sequencing of two model diatoms, *Phaeodactylum tricornutum* and *Thalassiosira pseudonana*, has offered the genetic basis for the biotechnology development of diatoms. The reconstruction of genome-scale metabolic models (GEMs) in diatoms has promoted metabolic engineering strategies to improve algal productivity. Genome engineering tools such as CRISPR (clustered regularly interspaced short palindromic repeats)/Cas (CRISPR associated system), transcription activator-like effector nucleases (TALENs), zinc-finger nucleases (ZFNs), microRNA (miRNA), and small interfering RNA (siRNA) are in rapid development to harness the genomic potential. For instance, the genome of diatom *P. tricornutum* could be efficiently edited by CRISPR/Cas9 system (Ng et al., 2017) and the TALEN-mediated system (Serif et al., 2017). Thus, diatoms have broad prospects in the production of value-added compounds with systems and synthetic biology tools enabling the characterization of many biosynthetic pathways and related genes. Nevertheless, the bottleneck of commercializing commodities like biomaterials (such as polyhydroxyalkanoates) and biofuels by microalgal cell factories lies in the biomass productivity in high-density culture. Optimizing photosynthetic efficiency (PE) may achieve high overall productivity as microalgae are considered efficient solar energy converters with metabolic flexibility. For compound production in the high-density culture of diatoms, it is feasible to utilize a two-phase culture, which possesses high biomass productivity under optimal conditions in the first stage and then accumulates value-added compounds such as EPA and fucoxanthin under stress conditions in the second stage, showing the potential for industrial applications (Yang and Wei, 2020; Parkes et al., 2021).

As a group of oil-producing algae, diatoms can be used as a source to produce biofuels and bioactive compounds such as fatty acids and carotenoids. The omega-3 polyunsaturated fatty acids such as docosahexaenoic acid (DHA) and eicosapentaenoic acid (EPA) from diatoms have critical biological functions for fetal growth and development based on their roles in maintaining the healthy function of the brain and retina (Marella and Tiwari, 2020). In addition, diatoms can produce non-native value-added compounds through heterologous expression systems, such as plant triterpenoids, monoclonal antibodies, and bioplastics (Butler et al., 2020; Lu et al., 2021). The traditional heterologous expression platforms like *Escherichia coli* and *Saccharomyces cerevisiae* have already been widely used. However, these classical expression systems require external organic carbon sources, limiting their carbon neutrality contributions (Hempel and Maier, 2012). Compared to other cell factories, such as bacteria, yeast, plants, and mammals (Table 1), diatoms have shown promise to become an ideal natural expression system due to their advantages of high efficiency, low cost, and the ability to synthesize complex value-added compounds. Green microalgae, such as the model species *Chlamydomonas reinhardtii*, have also been reported for the heterologous synthesis of value-added compounds. For comparison, diatoms could achieve stable genetic modifications with an available genetic toolbox and have a number of features; for example, *P. tricornutum* does have a free intracellular pool of GPP, while *C. reinhardtii* doesn’t accumulate GPP naturally (Fabris et al., 2020). Further, the rapid progress of diatom sequencing also provides new insights into diatom systems and synthetic biology.

**TABLE 1:** Advantages and disadvantages of different types of cell factories.

Platform	Advantages	Disadvantages	Example	References
bacteria	low cost; high growth rate; easy to perform	without post-translational modifications; require external nutrition	recombinant human insulin (Humulin®)	Walsh, (2018)
yeasts	have post-translational modifications	require external nutrition	humanized Ab (Herceptin®)	Liu et al., (2018)
plants	no external nutrition needed; low cost	occupy the land for crops; restricted by-laws; low growth rates and yields	recombinant taliglucerase alfa	Grabowski et al., (2014)
mammals	mature commercial applications; similar to humans; have post-translational modifications	high cost; low efficiency; susceptible to pathogens	recombinant c1- esterase inhibitor	van Veen et al., (2012)
diatoms	rapid biomass accumulation; have post-translational modifications	low efficiency of autotrophy at high density; transgene-silencing; fewer Synthetic biology tools	monoclonal antibodies	Hempel et al., (2017)

In this review, we specifically focus on 1) diatom systems biology, including genome sequencing and GEMs, 2) recent progress in PE optimization, and 3) native and non-native bioactive compounds and their biosynthetic pathways in diatoms manipulated by synthetic biology. Finally, we highlight challenges and discuss possible directions for developing algae-based value-added products.

## 2 Recent Progress in Diatom Systems Biology

*P. tricornutum* (Bowler et al., 2008) and *T. pseudonana* (Armbrust et al., 2004) have been used as two model diatoms for biological research due to their rapid growth and relatively small genome sizes. Their genome sequencing has incredibly advanced system biology development. Recently, more diatoms have been sequenced, with the complete genomes available, including *Fistulifera solaris* (Tanaka et al., 2015), *Skeletonema costatum* (Ogura et al., 2018), *Seminavis robusta* (Osuna-Cruz et al., 2020), and *Cyclotella cryptica* (Traller et al., 2016). Twenty-four whole-genome sequences from 21 diatom species are available to the public, according to the data retrieved from NCBI (the National Center for Biotechnology Information, United States) (as shown in Table 2). The genome sequencing results indicate that many species-specific genes are present in the diatom genome, and some diatom genes are obtained from bacteria by horizontal gene transfer (Falciatore et al., 2020; Bhattacharjya et al., 2021).

**TABLE 2:** Summary of sequenced diatom genomes.

Scientific name	GenBank	Modifier	Size (Mbp)	Year
*Asterionellopsis glacialis*	GCA_014885115.2	A3	66.88	2020
*Chaetoceros muellerii*	GCA_019693545.1	NMCA1316	37.74	2021
*Chaetoceros tenuissimus*	GCA_021927905.1	NIES-3715	41	2021
*Cyclotella cryptica*	GCA_013187285.1	CCMP332	171.1	2020
*Cylindrotheca fusiformis*	GCA_019693525.1	UTEX2084	48.25	2021
*Fistulifera pelliculosa*	GCA_019693425.1	UTEX661	30.16	2021
*Fistulifera solaris*	GCA_002217885.1	JPCC DA0580	49.74	2017
*Fragilaria radians*	GCA_900642245.1	/	98.38	2019
*Fragilariopsis cylindrus* CCMP1102	GCA_900095095.1	/	68.97	2016
*Fragilariopsis cylindrus* CCMP1102	GCA_001750085.1	CCMP1102	80.54	2016
*Halamphora sp.* AAB	GCA_004335955.1	AAB	29.55	2019
*Halamphora sp.* MG8b	GCA_004335815.1	MG8b	53.21	2019
*Licmophora abbreviata*	GCA_900291995.1	/	29.21	2018
*Nitzschia inconspicua*	GCA_019154785.2	hildebrandi	99.71	2021
*Nitzschia palea*	GCA_019593585.1	CPCC-160	41.16	2021
*Nitzschia putrida*	GCA_016586335.1	NIES-4239	47.13	2020
*Nitzschia sp.* Nitz4	GCA_013372465.1	Nitz4	27.23	2020
*Phaeodactylum tricornutum* CCAP 1055/1	GCA_000150955.2	CCAP 1055/1	27.45	2008
*Pseudo-nitzschia multistriata*	GCA_900660405.1	B856	56.77	2019
*Skeletonema costatum*	GCA_018806925.1	RCC75	51.09	2021
*Thalassiosira oceanica*	GCA_019693575.1	NMCA1005	83.51	2021
*Thalassiosira oceanica*	GCA_000296195.2	CCMP1005	92.04	2012
*Thalassiosira pseudonana* CCMP1335	GCA_000149405.2	CCMP1335	32.44	2009
*Thalassiosira sundarbana*	GCA_020086505.1	SBOTS_1isolate	52.89	2021

GEMs are the bottom-up description of cell metabolism based on the annotated genome and known metabolic reactions (Levering et al., 2016; Broddrick et al., 2019). They offer a whole-cell view of metabolic fluxes, act as powerful tools to discover the exceptional metabolic capabilities of cells and predict the internal metabolism of the organism under different conditions. Based on the whole life cycle, GEM development is usually divided into 4 phases: inception (the GEM model is firstly constructed based on an existing reference database), maturation (GEM is continuously updated as new information/knowledge becomes available), specialization (existing high-quality GEMs are tailored to specific strain cell lines), and amalgamation (several high-quality GEMs are wired together to form a multicellular model) (Seif and Palsson, 2021). The first diatom GEM was reconstructed for *P. tricornutum* (*i*LB1027_lipid), with 2,172 metabolites distributed in 6 compartments and 1,027 genes for 4,456 reactions, focusing on lipid metabolism (Levering et al., 2016). Later, the GEMs of *T. pseudonana* CCMP 1335 (*i*Thaps987 and *i*Tps1432) and polar diatom *Fragilariopsis cylindrus* were reported (Ahmad et al., 2020; Lavoie et al., 2020; van Tol and Armbrust, 2021). Previous studies also developed several strategies to improve the reconstruction efficiency of GEMs. For example, a standard sub-model chloroplast template (iGR774) with 774 genes, 788 reactions, and 764 metabolites could be incorporated into chloroplast-containing organisms with a minor modification (Bjerkelund Rokke et al., 2020). A novel software pipeline, Rapid Annotation of Photosynthetic Systems (RAPS), has been developed to create the first draft GEM of new species by using manual curation efforts of published models. The models produced by RAPS could capture more genes, showing more excellent capabilities to predict experimentally determined growth rates (Metcalf et al., 2020). By applying the GEMs, the physiological performance of diatoms can be modeled and predicted. After elevating CO_2_ levels, 137 reactions in eight different metabolic groups of *P. tricornutum* have fluxes rising, indicating pathways related to biomass production enhancement (Levering et al., 2017). However, this phenomenon was not observed under nitrogen limitation (Levering et al., 2017). The intercompartment reductant shuttles of *P. tricornutum* to transfer and consume additional light energy were deciphered under the combination of photo-physiology data and GEMs, supporting the prediction of photoprotective pathways for diatoms under different light intensities (Broddrick et al., 2019). A compartmentalized GEM for microalgae was reconstructed to reveal the metabolic pathway for chitosan and rhamnose synthesis, enabling metabolic engineering with synthetic biology tools (Juneja et al., 2016). Therefore, GEMs can provide insights into the metabolic information of biomass allocation in diatoms and build the foundation for synthetic biology development.

However, only three diatoms’ GEMs have been reported so far. A small proportion of genes are used in modeling (9.9% for *P. tricornutum*, 8.6% for *T. pseudonana*, and 3.9% for *F. cylindrus*). The reconstruction of diatoms’ GEMs is still in its early stages. Two bottlenecks of GEM reconstruction are maturation (Parts of the model need to be improved based on future research, and genes for newly discovered metabolic pathways also need to be added to the model) and content removal (remove incorrect or redundant parts).

## 3 Synthetic Biology for Photosynthetic Efficiency and Biomass Productivity Enhancement in Diatoms

### 3.1 Explorations for Photosynthetic Efficiency Developments

To tackle the reduction of photosynthetic efficiency at high-density culture, strain engineering for PE is needed to accumulate more value-added compounds. More than half of the energy is lost when the electron transport chain (ETC) is saturated due to the fast rate of photon capture, which may be 100 folds greater than the downstream rate of photosynthetic electron flow (Perrine et al., 2012). Therefore, the gap between currently achievable and maximum thermodynamic efficiency for photosynthesis is substantial. Accordingly, physical and biotechnological methods could reduce energy loss during photosynthesis in some microalgae, such as diatoms.

In photonic fields, a recent study showed that an ideal arrangement of timing in photosynthetic pathways achieved by pulsed light is the key to increasing algal PE (Sforza et al., 2012; Zarmi et al., 2020). Apart from that, the interaction between ocean acidification and different light environments presents another insight for PE enhancement (Briggs and Carpenter, 2019). For diatoms, the photonic structures of their frustules have also inspired photonics and nanotechnology studies. The role of frustules in trapping light was studied and applied in dye-sensitized solar cells (DSSCs) (Toster et al., 2013). The cellular light field could be altered by the optical properties of the nanostructure of frustule valves. Thus, in centric diatoms *Coscinodiscus granii*, PE was enhanced by photon trapping and scattering (Goessling et al., 2018). Therefore, developing biotechniques to synchronize photonics with biological timescales and modifying optical properties of frustules could be promising for PE enhancement.

In biotechnological fields, in addition to conventional culture condition optimization, such as a study of pH effects on microalgae (Costache et al., 2013), synthetic biology has emerged for PE enhancement through comparative phylogenomic analyses, proteomic studies, and transcriptomic analyses. In addition, transcriptome sequencing and annotation give clues to target genes for PE and biomass productivity enhancement (Xi et al., 2021). However, photosynthetic genes and relevant pathways in many microalgae are underexplored or restrained to certain model species like *C. reinhardtii,* which possesses a genome-wide library of mutants (Li et al., 2019), as half of the protein-coding genes for photosynthetic organisms remain uncharacterized (Krishnakumar et al., 2015). That dilemma mainly lies in its genetic diversity and variability (Fu et al., 2019; Butler et al., 2020). However, the recent developments in PE are still encouraging, as PE in diatoms has been studied at the cellular and subcellular level (Yan et al., 2018; Uwizeye et al., 2021).

One study on photosystem (PS) structure in diatoms suggested that light utilization could be improved *via* interconnected subunits to ensure the equilibration of electron carriers (Flori et al., 2017). Over the years, many studies focused on diatom PS II-light-harvesting antenna complex (PSII-FCPII) composing of PS and fucoxanthin chlorophyll a/c proteins (FCP) as light-harvesting antenna complex (LHC) for its structural basis (Nagao et al., 2019), light-harvesting ability (Nagao et al., 2014a), and energy transfer mechanism (Nagao et al., 2014b). With the elucidation of the PSII-FCPII supercomplex, the structural basis for detailed mechanisms regulating energy transfer and quenching in diatoms was deciphered (Wang W. et al., 2020). The structure of the PSI-FCPI supercomplex was also reported in diatom *Chaetoceros gracilis* consisting of cores, antenna subunits, and pigments (Xu et al., 2020). These results revealed the structural basis of PE and provided a foundation for further study of PE by reconstructing or replacing PS subunits. The process of exploring PE has always been accompanied by the study of the inhibition of photosynthesis. One research on allelochemicals from the toxic dinoflagellate revealed the negative effect of membrane damage to the PE of diatom *Chaetoceros muelleri* (Long et al., 2021). Similarly, recent studies on diatom *P. tricornutum* have shown that plasma membrane-type aquaporins (AQPs) were related to the efflux of NH_3_, which promoted non-photochemical quenching (NPQ) (Matsui et al., 2018). Moreover, the research on *Synedra sp.* showed PE decreased under the structural damage of thylakoids (Yao et al., 2019). Once the connections between these factors and the inhibition of PE are clear, one can engineer a more efficient apparatus for microalgal photosynthesis.

Currently, optimization of inorganic carbon utilization has also shown promise for PE enhancement. The carbon concentrating mechanisms (CCMs) play a vital role in concentrating CO_2_ to ribulose-1, 5-bisphosphate carboxylase oxygenase (Rubisco). It consists of Ci transportation, carboxysomes, and CAs (Barati et al., 2021). A recent study has demonstrated that related genes such as HLA3 (Yamano et al., 2015) and LCI1 (Kono et al., 2020) are essential for CCM functioning. It is also feasible to genetically modify CCM (Gao et al., 2015). Carbonic anhydrases (CAs), one of the critical components in CCMs, have received extensive studies. In 2014, δ-class carbonic anhydrase (TweCA) and a novel CA called CDCA1 were discovered in the diatom *Thalassiosira weissflogii*, demonstrating the relationship between photosynthesis and metal ions in water and the potential use of metalloenzyme to capture CO_2_ (Del Prete et al., 2014; Alterio et al., 2015). The genome sequencing of two model diatoms, i.e., *P. tricornutum* and *T. pseudonana*, enabled the elucidation of genes related to their CCMs, indicating that CA played a crucial role in CCM for inorganic carbon acquisition (Hopkinson et al., 2016). More CAs were discovered, such as Pt43233. It was a novel θ-type CA essential for PE in *P. tricornutum,* which is sited in the lumen of thylakoid, demonstrating direct usage of the pH gradient to provide CO_2_ to the Calvin cycle (Kikutani et al., 2016). CCMs in some diatoms involved an external carbonic anhydrase (CA (ext)) that was influenced by external CO_2_ concentration (Smith-Harding et al., 2017). CCMs exhibited great diversity and were regulated mainly by the LysR family of transcriptional regulators, while CCMs in diatoms were related to pyrenoids (Tomar et al., 2017). Research on the accumulation of purple acid phosphatases (PAPs) showed that PAP1 promoted growth and photosynthesis in *P. tricornutum* by modifying carbon flux and phosphate acquisition (Wang et al., 2021). One recent study identified distinct carbon flux patterns supporting higher PE in microalgae than in C3 and C4 plants (Treves et al., 2022).

### 3.2 Synthetic Biology Tools for Photosynthetic Efficiency and Biomass Productivity Enhancement

Photosynthetic pigments, consisting of three major classes, are responsible for harvesting light energy from photosynthetically active radiation (PAR) ranging from 400 to 700 nm in green plants and most microalgae, while broader absorption spectra could be achieved by varied chlorophyll species that were discovered naturally in several bacteria with more remarkable light-harvesting ability (Naduthodi et al., 2021). Although the detailed genes involved remain unclarified, it’s feasible to enhance PE by expanding the light absorption spectrum with non-native pigments. An approach for PE enhancement termed intracellular spectrum recompositioning (ISR) in diatoms also showed promising results with a 50% increase in PE and biomass productivity (Fu et al., 2017). In the ISR study, a green fluorescent protein (GFP) was expressed and engineered in the model diatom *P. tricornutum* to shift excessive blue light to green light intracellularly, which optimized light distribution and absorption by heterologous counterparts (Fu et al., 2017). One recent study aimed at non-genetic tailoring light-harvesting by adding a molecular dye (Cy5) to fix the orange gap of absorption spectrum in the diatom *Thalassiosira weissflogii*, demonstrating an enhancement of PE and biofuel generation (Leone et al., 2021). The study on the diatom *Corethron hystrix* under high UV stress found the gene expression of PSII-related proteins D1, CP43, and CP47 was suppressed, indicating a genetic engineering clue for PE enhancement under high UV threat through a unique protective mechanism against UV exposure (Read et al., 2019). NPQ, termed non-photochemical quenching, represents the light energy lost in photosynthesis due to inefficiency caused by the wrong size of the LHC related to chlorophyll pigments. Antenna size optimization was achieved via several approaches, such as RNAi technology (Barry et al., 2015) and mutagenesis attained by UV and ethyl methanesulfonate (EMS) mediation (Cazzaniga et al., 2014; Shin et al., 2016). Recent studies have shown the possibility to adjust LHC sizes dynamically by proteins such as chloroplast signal recognition particle (CpSRP) developed for antenna size reduction (Naduthodi et al., 2021).

The key enzymes in Calvin–Benson–Bassham (CBB) cycle play a vital role in rate-limiting steps. The shortcomings of Rubisco, an enzyme allocating the carbon flux in the CBB cycle, serve as an engineering target in genetic modifications for PE. Over-expression of this enzyme or its activase (Ng et al., 2017) is a possible way to enhance its catalytic ability. In recent years, protein engineering and the genome editing toolbox have boosted the crucial progress on Rubisco engineering for better performance by point mutations and genetic modification (Sharwood, 2017). In addition, the native Rubisco enzyme could be replaced with heterologous expression of superior variants identified in systems biology studies (Satagopan et al., 2019). For example, diatoms evolved with Rhodophyte Form 1D Rubisco, which is more suitable in CCM to achieve high carboxylation rates under high O_2_/CO_2_ ratios (Rickaby and Eason Hubbard, 2019). However, these variants are usually only available with a specific supplement of activates or chaperone proteins as the heterologous proteins may also disrupt subcompartments with unique binding motifs (Meyer et al., 2020). Compared to engineered Rubisco enzyme variants, the heterologous expression for homodimeric Rubisco may be an ideal option in microalgae. Following this approach, other enzymes of the CBB cycle were considered. For instance, the overexpression of sedoheptulose bisphosphatase (SBP) (Yang et al., 2017), fructose 1,6-bisphosphate aldolase (FBA) (Ogawa et al., 2015; Yang et al., 2017), and ribulose-5-phosphate kinase (PRKase) (Ng et al., 2017) activated intermediate regeneration and energy reallocation in CBB cycle. The efficiency of the CBB cycle relies largely on CCM. A study integrated the individual components of *C. reinhardtii* CCM into the chloroplast of tobacco plants (C3 plant) by inserting cDNAs encoding the protein (CAH3 and LCIA), demonstrating increased PS II efficiency (up to 18%) as well as more accumulation of biomass in the newly generated transgenic strains (Nolke et al., 2019). A further study proved that when *C. reinhardtii* LCIA (CrLCIA) gene was heterologously expressed in *Nannochloropsis salina* CCMP1776, the activity of Ci and CA were enhanced, resulting in an increase in biomass productivity up to two folds (Vikramathithan et al., 2020). These studies on green microalgae indicate that the heterologous expression of CCM components is the potential for PE enhancement in diatoms. But due to the complexity of the steps and pathways involved, the engineering scheme for this mechanism is still in the early stage and needs further study.

**FIGURE 1:**
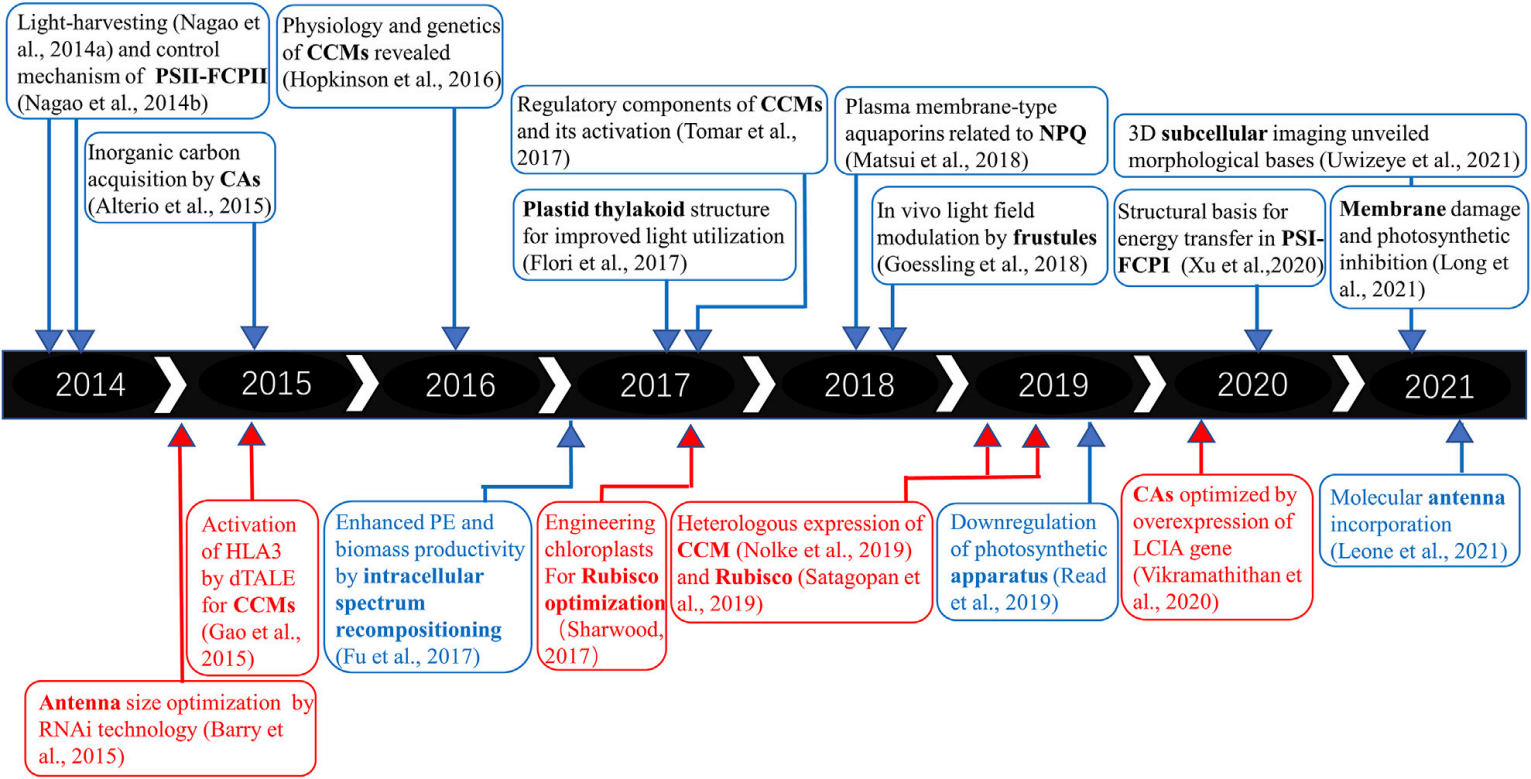
Timeline and basic and applied research trends on microalgal PE and biomass productivity. The upper panel refers to fundamental research on PE in diatoms, and the lower panel refers to practical applications of synthetic biology tools. The red part represents green microalgae, while the blue region represents diatoms.

Some green microalgae, such as *C. reinhardtii,* are model organisms for physiological study, but the difficulty and inefficiency in heterologous gene expression limit the research development. In that case, diatoms, as one of the most successful photosynthetic groups (Hippmann et al., 2022), have caught great attention from the science community in recent years for their genetic modification potential. Figure 1 summarizes the development of green microalgae and diatoms in PE enhancement. Great possibilities still lie in improving PE and biomass productivity in diatoms. The exploration of photosynthesis raises insight into fields beyond. By testing carbon dioxide- or bicarbonate-capturing efficiencies in open ponds, microalgae demonstrated their strong ability to fix bicarbonate *via* photosynthesis, thus thought to be well suited for carbon capture and sequestration (Sayre, 2010). Bioenergy production combined with carbon sequestration (BECCS) is considered a key strategy to deal with the global threat of climate change caused by greenhouse gases in the context of sustainable development goals (SDGs) (Barry et al., 2015). Meanwhile, the enhancement of photosynthetic efficiency also indicates the potential for microalgae in advanced wastewater treatment (Qi et al., 2021)and photovoltaic devices (Cho et al., 2019). To overcome the drawbacks of natural and artificial photosynthesis, a semi-artificial photosynthetic system was developed to efficiently generate solar fuels and chemicals (Kornienko et al., 2018).

## 4 Synthetic Biology for Value-Added Products in Diatoms

### 4.1 Production of Homologous Compounds

#### 4.1.1 Fucoxanthin

Fucoxanthin (Fx) is an essential marine carotenoid that widely exists in microalgae, functioning as an anticancer, antibacterial, and hypoglycemic agent (Wang J. et al., 2019; Karpiński and Adamczak, 2019). Currently, Phaeophyta (brown algae) is the primary source of Fx. But due to its low yield, low quality, and high cost, it is difficult to meet market demand. Alternatively, the marine diatom *P. tricornutum* has more than 100 times the Fx content of Phaeophyta. Thus it has been considered a promising Fx commercial producer (Yang and Wei, 2020). Fx with biological activity plays a vital role in diatom growth. It exists together with chlorophylls as fucoxanthin–chlorophyll protein (FCP), which is crucial for the light-harvesting complex (Gelzinis et al., 2015; Wang W. et al., 2019). FCP enables diatoms to absorb light in a broad spectrum, contributing to energy transfer and dissipation in photosynthesis (Wang W. et al., 2019).

**FIGURE 2:**
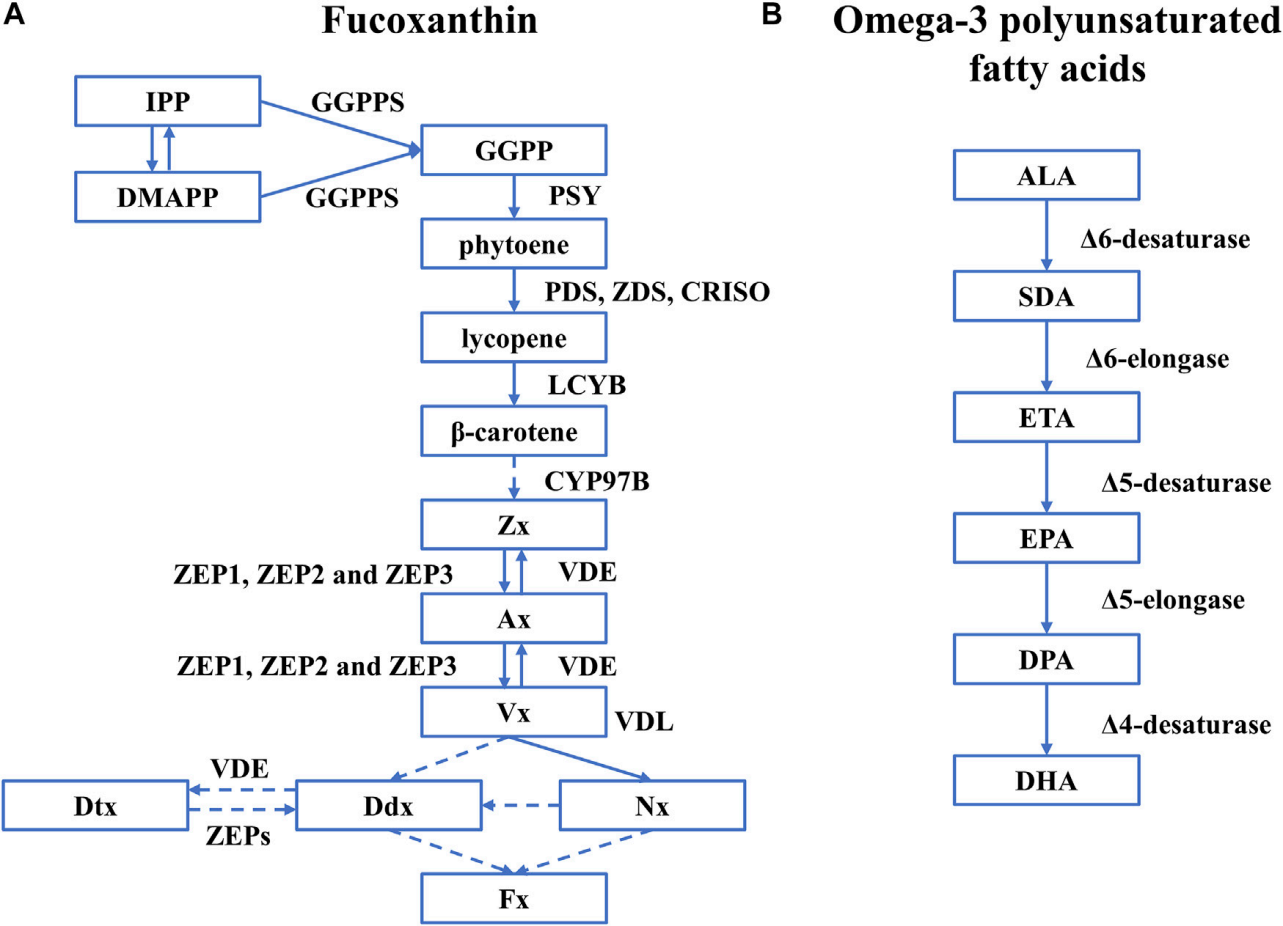
The biosynthetic pathway of Fx and omega-3 polyunsaturated fatty acids in diatoms. Rectangles represent intermediates, and arrows represent biosynthetic pathways and active enzymes. **(A)** Fucoxanthin. The figure was adapted from a previous publication (Manfellotto et al., 2020). A dashed line represents the unknown biosynthetic process. **(B)** omega-3 polyunsaturated fatty acids. The figure was adapted from a previous publication (Sayanova et al., 2017) and only covers the pathway of omega-3 polyunsaturated fatty acids. Abbreviations: IPP, isopentenyl pyrophosphate; DMAPP, dimethylallyl diphosphate; PSY, phytoene synthase; GGPPS, geranylgeranyl pyrophosphate synthase; GGPP, geranylgeranyl diphosphate; PDS, phytoene desaturase; ZDS, ζ-carotene desaturase; CRISO, carotenoid isomerase; LCYB, lycopene β-cyclase; Zx, zeaxanthin; ZEP1, ZEP2, and ZEP3, three types of Zea epoxidase; VDE, violaxanthin de-epoxidase; Vx, violaxanthin; VDL, violaxanthin de-epoxidase-like proteins; Nx, neoxanthin; Ddx, diadinoxanthin; Dtx, diatoxanthin.

The biosynthesis of Fx in diatoms has been extensively studied but not fully understood (Figure 2A). It is generally agreed that the synthesis of Fx starts from isopentenyl pyrophosphate (IPP) and its isomer dimethylallyl diphosphate (DMAPP) that are catalyzed by geranylgeranyl pyrophosphate synthase (GGPPS) to generate geranylgeranyl diphosphate (GGPP) (Fraser Paul et al., 2002; Cazzonelli and Pogson, 2010). Then the GGPP is converted to phytoene by phytoene synthase (PSY), which is the rate-determining step (Cazzonelli and Pogson, 2010). With phytoene desaturase (PDS), ζ-carotene desaturase (ZDS), and carotenoid isomerase (CRISO), phytoene is converted to lycopene, which is further catalyzed by lycopene β-cyclase (LCYB) to produce β-carotene. Cytochrome P450-type carotene hydroxylases (CYP97A/B/C) are believed to be involved in synthesizing the subsequent zeaxanthin (Zx) in microalgae. However, only the gene encoding CYP97B has been identified in *P. tricornutum* (Cui et al., 2019). Then, Zx is catalyzed to generate violaxanthin (Vx) by zeaxanthin epoxidase (ZEP1, ZEP2, and ZEP3) through intermediate metabolites antheraxanthin (Ax), which is further converted to Zx by violaxanthin de-epoxidase (VDE), forming the violaxanthin cycle (Eilers et al., 2016b). However, the next step to generate Fx is not fully understood with two hypothetical paths: 1) Vx is catalyzed by violaxanthin de-epoxidase–like (VDL) proteins to generate neoxanthin (Nx) as the precursor of Fx and diadinoxanthin (Ddx) (Dautermann et al., 2020), 2) Vx transfers to Ddx as the precursor of Fx and diatoxanthin (Dtx) (Bertrand, 2010; Dambek et al., 2012).

It has been found that 1-deoxy-D-xylulose 5-phosphate synthase is necessary to generate IPP for initiating Fx biosynthesis. With the overexpression of the gene that encodes 1-deoxy-D-xylulose 5-phosphate synthase, the content of Fx increased up to 2.4 folds (Eilers et al., 2016a). Phytoene synthase (PSY) is one of the most important enzymes involved in the rate-determining step. The PSY gene fused with the enhanced green fluorescent protein (eGFP) gene was successfully introduced into *P. tricornutum* and overexpressed, resulting in an increase of Fx content by about 1.45 folds in the stationary phase and about 1.19 folds in total compared with the wild type (WT) (Kadono et al., 2015). A new technique for high-throughput screening of high-Fx-content diatom mutants using flow cytometry was invented and effectively separated the strains with higher Fx content, which was 25.5% higher than that in the WT (Fan et al., 2021). The simultaneous overexpression of three genes, violaxanthin de-epoxidase (Vde), Vde-related (Vdr), and zeaxanthin epoxidase 3 (Zep3) genes, resulted in a four-fold increase in Fx content in *P. tricornutum* (Manfellotto et al., 2020).

#### 4.1.2 Lipids

Diatoms can produce a variety of lipids, including triacylglycerol (TAG) and other neutral lipids, which makes them a potential source for biofuels. In addition, diatoms can produce biologically active omega-3 polyunsaturated fatty acids such as EPA and DHA that support the growth of organisms (Li-Beisson et al., 2019). They can be used to promote the learning and memory ability of the brain, inhibit the proliferation of some tumor cells, and improve glucose and lipid metabolism disorders (Zhang et al., 2019). EPA and DHA are mainly extracted from marine fish that can quickly accumulate harmful substances. Given that the ecological crisis caused by the overfishing of marine animals is also grievous, diatoms are considered a healthy and sustainable substitute (Fu et al., 2022).

The synthesis of omega-3 polyunsaturated fatty acids starts from α-linolenic (ALA, C18:3) obtained by the saturated fatty acids (C18:0) with the Δ9-, Δ12-, and Δ15-desaturases. EPA and DHA are synthesized with a series of desaturases and elongases, including Δ6-, Δ5- and Δ4-desaturases, and Δ6- and Δ5-elongases (Sayanova et al., 2017). The specific synthetic route is as follows (Figure 2): 1) As a precursor of omega-3 LC-PUFAs, α-linolenic (ALA, C18:3) is used to generate stearic acid (SDA, C18:4) by the action of Δ6-desaturase, and then synthesize eicosatetraenoic acid (ETA, C20:4) under the catalysis of Δ6-elongase. 2) ETA is converted to eicosapentaenoic acid (EPA, C20: 5) by Δ5-desaturase. 3) EPA is first elongated to docosapentaenoic acid (DPA, C22:5) by Δ5-elongase and then desaturated by a Δ4-desaturase to produce docosahexaenoic acid (DHA, C22:6) (Zulu et al., 2018).

Abiotic stress and genetic modification approaches have improved diatoms’ productivity for value-added compounds. Abiotic stresses, such as light, salinity, pH, and temperature stress, can increase the content of metabolites but reduce the biomass productivity of diatoms, leading to a decrease in overall yield. For example, under low salinity conditions, the EPA content in the arctic diatom *Attheya septentrinalis* increased, while its growth rate dropped significantly compared with that under the natural seawater condition (Steinrücken et al., 2018). In addition, the lipid content in *P. tricornutum* increased under high pH stress, while its carotenoid content increased under low pH stress, although their growth rates decreased under both pH stress conditions (Mus et al., 2013). On the contrary, genetic engineering can improve the content of bioactive compounds without reducing biomass production, which is beneficial to the productivity of target products. By inserting an additional endogenous Δ6 fatty acid desaturase gene (PtD6) into the *P. tricornutum* genome, the overexpression of PtD6 resulted in an increase in total lipids and EPA (Zhu et al., 2017). Similarly, overexpression of the Δ5-desaturase gene (PtD5b) in *P. tricornutum* increased the EPA content by 58% and also produced 0.44 mg/g DHA, which was not observed in the wild type (Peng et al., 2014). Under the synergistic effect with Δ5-desaturase (PtD5b) and malonyl CoA-acyl carrier protein transacylase (MCAT), the total lipid content was increased by 2.61 folds without impairing the growth of *P. tricornutum*, and the content of EPA and DHA also increased at the same time (Wang et al., 2017). One diacylglycerol acyltransferases (DGAT2B) gene was introduced and co-expressed with the Δ5-elongase (OtElo5) gene in *P. tricornutum*, leading to a higher content of DHA (3.8–8.4% of dry biomass) compared with the WT (1.8% of dry biomass) (Haslam et al., 2020).

Meganucleases (MNs) and transcription activator-like effector nucleases (TALENs) are practical tools for gene editing, such as gene knockout and gene knock-in. Using these technologies to disrupt the carbohydrate storage gene UDP-glucose pyrophosphorylase (UGPase) in *P. tricornutum* resulted in a 45-fold increase in TAG content in nutrient replete culture and a 3-fold increase in nitrogen-depleted culture (Daboussi et al., 2014). With the development of gene-editing technology, CRISPR/Cas9 has been applied to diatoms, which can realize gene editing quickly and efficiently at a low cost but with high accuracy (Nymark et al., 2017). With the aid of this technique, up to five genes in *P. tricornutum* were edited successfully, increasing total lipid content dramatically (Sharma et al., 2021).

#### 4.1.3 Sterols

Sterols, a type of triterpenoid, are the most abundant isoprenoids produced by diatoms, effective for cholesterol-lowering and anti-inflammation (Aldini et al., 2014). Sterols function as regulators of cell membrane dynamics in diatoms, but their synthetic pathways are not fully deciphered. Overexpression of HMGR (3-hydroxy-3-methyl glutaryl CoA reductase) in *P. tricornutum* increased the levels of sterol intermediates such as squalene, cycloartenol, and obtusifoliol, but the total sterol content did not change, indicating that there is a mechanism controlling the overall content in diatoms (Jaramillo-Madrid et al., 2020). Recently, independent genes distinct from other diatom species have been found in the marine diatom *Rhizosolenia setigera*, which encode two isopentenyl diphosphate isomerases (RsIDI1 and RsIDI2) and squalene synthase (RsSQS) that catalyze the synthesis of isoprenes. The sequence analyses suggested that these genes might come from a secondary gene fission event (Ferriols et al., 2017).

### 4.2 Production of Heterologous Compounds

**FIGURE 3:**
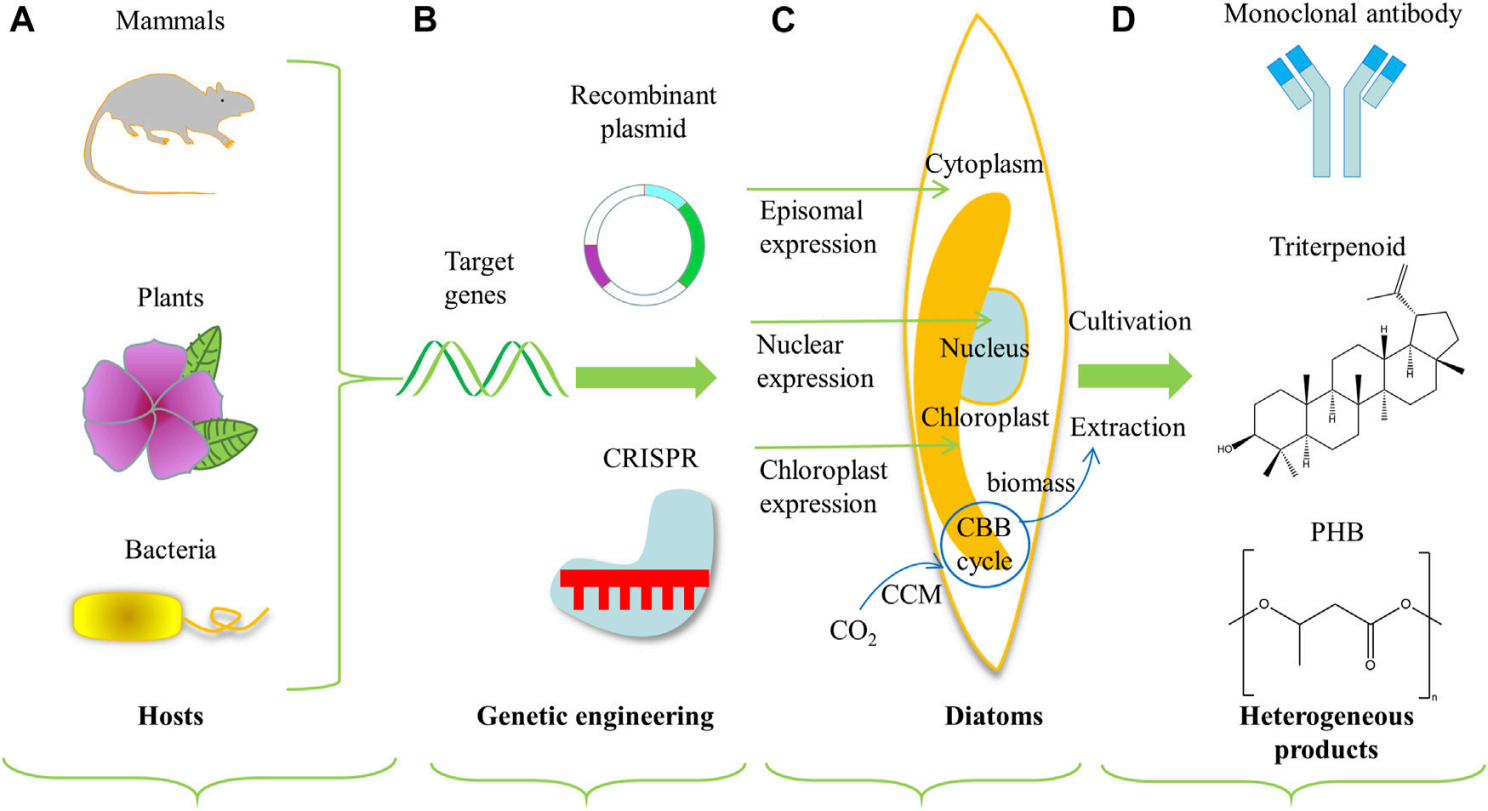
Schematic diagram of engineering diatoms as cell factories for heterologous production of value-added compounds. Heterologous expression system includes **(A)** the selection of target source genes, **(B)** conventional genetic engineering or genome engineering technologies like CRISPR, **(C)** DNA transformation and expression locus in diatoms, and **(D)** production of value-added compounds (D'Adamo et al., 2019). The part with blue cycle and arrows represents the framework for inorganic carbon utilization. Abbreviations: CBB, Calvin–Benson–Bassham cycle; CCM, carbon concentrating mechanism; PHB, Poly-β-hydroxybutyrate.

Diatoms have also been developed and engineered as photosynthetic cell factories to produce heterologous value-added bioactive compounds (as shown in Figure 3). When diatoms express heterologous compounds, the native product in diatoms (such as TAGs) can be extracted simultaneously to reduce costs further (D'Adamo et al., 2019). Heterologous compounds expressed in diatoms were summarized in Table 3, including plasmid design and transformation methods.

**TABLE 3:** Heterologous compounds expressed in diatoms.

Heterologous compounds	Hosts	Promoter	Selective makers	Transformation method	Yield	References
Human IgG antibody^1^	*P. tricornutum*	nitrate reductase promoter	GFP	microparticle bombardment	1.6 mg/L	Hempel et al., (2011b)
Human IgG antibody^1^	*P. tricornutum*	nitrate reductase promoter	GFP	microparticle bombardment	2.5 mg/L	Hempel and Maier, (2012)
Human IgG antibody^2^	*P. tricornutum*	nitrate reductase promoter	GFP	microparticle bombardment	1.3 mg/L	Hempel et al., (2017)
Bisabolene	*F. solaris*	GAPDH gene promoter	nptII	microparticle bombardment	0.81 ± 0.22 mg/L	Tanaka et al., (2021)
Lupeol	*P. tricornutum*	*FCPA/LHCF1* promoter	*ble* ^r^ and *nat* ^r^	microparticle bombardment	0.1 mg/L	D'Adamo et al., (2019)
Geraniol	*P. tricornutum*	upstream of Phatr3_J49678, Phatr3_49202, Phatr3_J21569, Phatr3_J1 35766 and Clp1p	mVenus (YFP)	bacterial conjugation	0.309 mg/L	Fabris et al., (2020)
Polyhydroxybutyrate (PHB)	*P. tricornutum*	nitrate reductase promoter	eGFP	microparticle bombardment	10.6% of dry cell weight	Hempel et al., (2011a)

ble^r^, zeocin resistance gene marker; eGFP, enhanced green fluorescent protein; FCPA/LHCF1, fucoxanthin chlorophyll a/c binding protein A promoter; GAPDH, glyceraldehyde 3-phosphate dehydrogenase; GFP, green fluorescent protein; natr, nourseothricin resistance selection marker; nptII, neomycin phosphotransferase gene; YFP, yellow fluorescent protein; 1the human monoclonal IgG antibody CL4mAb against the Hepatitis B surface antigen (HBsAg); 2the human monoclonal IgG antibody against pathogenic Marburg virus nucleoprotein.

#### 4.2.1 Recombinant Therapeutic Proteins

Due to specificity in targeting and modulating disease-related pathways, recombinant biologics have been widely used to treat various diseases (Dehghani et al., 2020). The vast majority of recombinant medical proteins are glycoproteins, implying that complex N-glycosylation and disulfide bond formation are necessary for adequately folding glycoprotein structures and achieving glycoprotein function. It is challenging to produce safe and qualified recombinant therapeutic protein products with prokaryotic cells by conventional heterologous expression. In addition, the high cost of mammalian cells limits their applications in developing and less-developed countries. With the natural advantages of photosynthetic autotrophy, such as fast reproduction and low cost, diatoms also have a complete post-translational expression pathway, which can directly secrete the hetero-expressed proteins into the medium. This feature reduces the complexity and cost of subsequent separation and purification in diatoms (Specht and Mayfield, 2014). Heterologous expression of recombinant proteins and antimicrobial peptides has also been reported in green microalgae. A single chain antibody that recognizes B-cell surface molecule CD22 and a chimeric immunotoxin gene contained the hinge and CH2 and CH3 domains of a human IgG1 were expressed in chloroplasts of *C. reinhardtii* (Tran et al., 2013). Antimicrobial peptide piscidin-4 gene with optimized codon was expressed in chloroplasts of the freshwater green microalga *Haematococcus pluvialis* (Wang K. et al., 2020).

Recombinant pharmaceutical proteins synthesized by microalgae mainly include monoclonal antibodies (mAbs), immunotoxins, human cytokines, recombinant hormones, and recombinant vaccines. The first recombinant proteins reported are the monoclonal IgG1/kappa antibody CL4mAb and the Hepatitis B surface antigen (HBsAg) subtype adr expressed in the *P. tricornutum*, resulting in 8.7% CL4mAb and 0.7% HBsAg of the total soluble protein yielded in the endoplasmic reticulum (ER). Antibody accumulation does not impair the growth rate and cell viability of *P. tricornutum*, and antibody expression levels do not decline over 1 year of testing, which is critical for large-scale industrial production (Hempel et al., 2011b). Similarly, two studies successfully expressed a human IgG antibody against the Hepatitis B Virus surface protein and monoclonal IgG antibodies against the nucleoprotein of Marburg virus in *P. tricornutum* (Hempel and Maier, 2012; Hempel et al., 2017).

#### 4.2.2 Heterologous Terpenoids

Terpenoids can be divided into monoterpenes (C10), sesquiterpenes (C15), diterpenes (C20), disesquiterpenes (C25), triterpenes (C30), and tetraterpenes (C40) according to the number of isoprene units in the molecule (Ma et al., 2021). Terpenoids are natural compounds widely used in the pharmaceutical industry, such as anticancer and antimalarial applications. But many terpenoids, such as triterpenes, are found in low concentrations in living organisms, increasing production costs due to purification complexity and limiting their commercial application (Ma et al., 2021). *E. coli* and *S. cerevisiae* are usually selected as the heterologous hosts for terpenoid production based on their rapid growth and clear metabolic pathways. However, diatoms are more closely related to plants than bacteria or yeast, meaning that cell environments of diatoms are more favorable to the synthesis of plant-derived terpenoids. Diatoms contain both the cytosolic mevalonate pathway (MVA) and the chloroplastic non-mevalonate pathway (MEP) to develop IPP, which is an essential precursor of the sterol metabolic pathway (Fabris et al., 2020). Moreover, the flux towards isoprenoids in microalgae may be higher than in other organisms (Lauersen, 2019). However, the unknown regulation and underlying mechanism of the isoprenoid pathway in diatoms affect further investigations of heterologous terpenoid production.

Intron-mediated enhancement is a phenomenon in many eukaryotes, increasing transcription speed, mRNA stability, and protein translation efficiency when introns are present (Shaul, 2017). By introducing this strategy, the native glyceraldehyde 3-phosphate dehydrogenase (GAPDH) intron was inserted into the coding sequence of *Abies grandis* bisabolene (a representative sesquiterpene) synthase gene to realize heterologous expression in the diatom *Fistulifera solaris* (Tanaka et al., 2021).

Geraniol is a plant monoterpenoid, the main component of rose essential oil. It is formed by the direct conversion of geranyl diphosphate (GPP) by the enzyme geraniol synthase. Unlike the cell factories that naturally do not accumulate GPP, such as yeast, *E. coli*, and *C. reinhardtii*, the marine diatom *P. tricornutum* has an intracellular pool of GPP but has no genes for terpene synthases (Jaramillo-Madrid et al., 2019; Fabris et al., 2020). These properties indicate no endogenous monoterpenoid biosynthetic pathways to compete for heterologous production of monoterpenoids. A full-length coding sequence of *Catharanthus roseus* geraniol synthase was introduced into *P. tricornutum* using an episomal expression strategy with episomes delivered into the diatom nuclei by bacterial conjugation (Fabris et al., 2020). The target gene was expressed in the cytoplasm, and the production of monoterpenoid geraniol reached 0.309 mg/L, a similar level as that achieved by yeast engineering (Fabris et al., 2020).

Lupeol is a plant triterpenoid with antioxidant, anti-inflammatory, and tumor-suppressive effects. It can be synthesized from 2,3-oxidosqualene precursors under the catalysis of oxidosqualene cyclases. In the plant sapogenin biosynthetic pathway, lupeol can be oxidized into betulinic acid under the catalysis of cytochrome P450 and NADPH reductase. Two lupeol synthase coding sequences from *Lotus japonicus* and *Arabidopsis thaliana* were successfully integrated into the *P. tricornutum* nuclear genome. Later, *Medicago truncatula* CYP716A12, a cytochrome P450-dependent monooxygenase, and the *Medicago truncatula* cytochrome P450 reductases (MtCPR) were expressed in *P. tricornutum* to synthesize betulinic acid (D'Adamo et al., 2019). Lupeol is harmless to host cells and may be secreted into the medium, which has inherent advantages in downstream extraction at the industrial level (D'Adamo et al., 2019). This study is more complex than other reports of modified diatoms, involving relatively complex material reactions and pathways. The stability of heterologous genes and their impact on the original metabolism should be considered when the complexity of strains increases (Ng et al., 2020).

#### 4.2.3 Bioplastics

The vast majority of plastic made from petroleum is challenging to degrade and lacks a complete recycling process. Most plastics are discharged into the sea after use and become microplastics gradually, which have detrimental effects on human health and the environment. Biodegradable bioplastics can effectively reduce plastic pollution and carbon emissions (Park and Lee, 2022). However, bioplastics made from land crops require large amounts of arable land, fertilizers, and freshwater. Among many bioplastics, polyhydroxybutyrate (PHB) produced in bacteria and cyanobacteria has attracted much attention due to its good mechanical, heat resistance, and biocompatibility properties (Hempel et al., 2011a; Sirohi et al., 2021). The entire PHB pathway of *Ralstonia eutropha* H16, which has three enzymes, PhaA (ketothiolase), PhaB (acetoacetyl CoA reductase), and PhaC (PHB synthase), has been expressed in the cytoplasm of *P. tricornutum*. However, the study of PHB-producing microalgae is still in its infant stage, and the metabolic pathway of PHB production in diatoms is not fully understood (Sirohi et al., 2021). Although diatoms synthesize PHB at a rate of 100 times faster than plant expression systems, they still cannot compete with the soil bacterium *R. eutropha,* which has been commercially used as the PHB producer for many years (Hempel et al., 2011a).

## 5 Concluding Remarks and Future Perspectives

**FIGURE 4:**
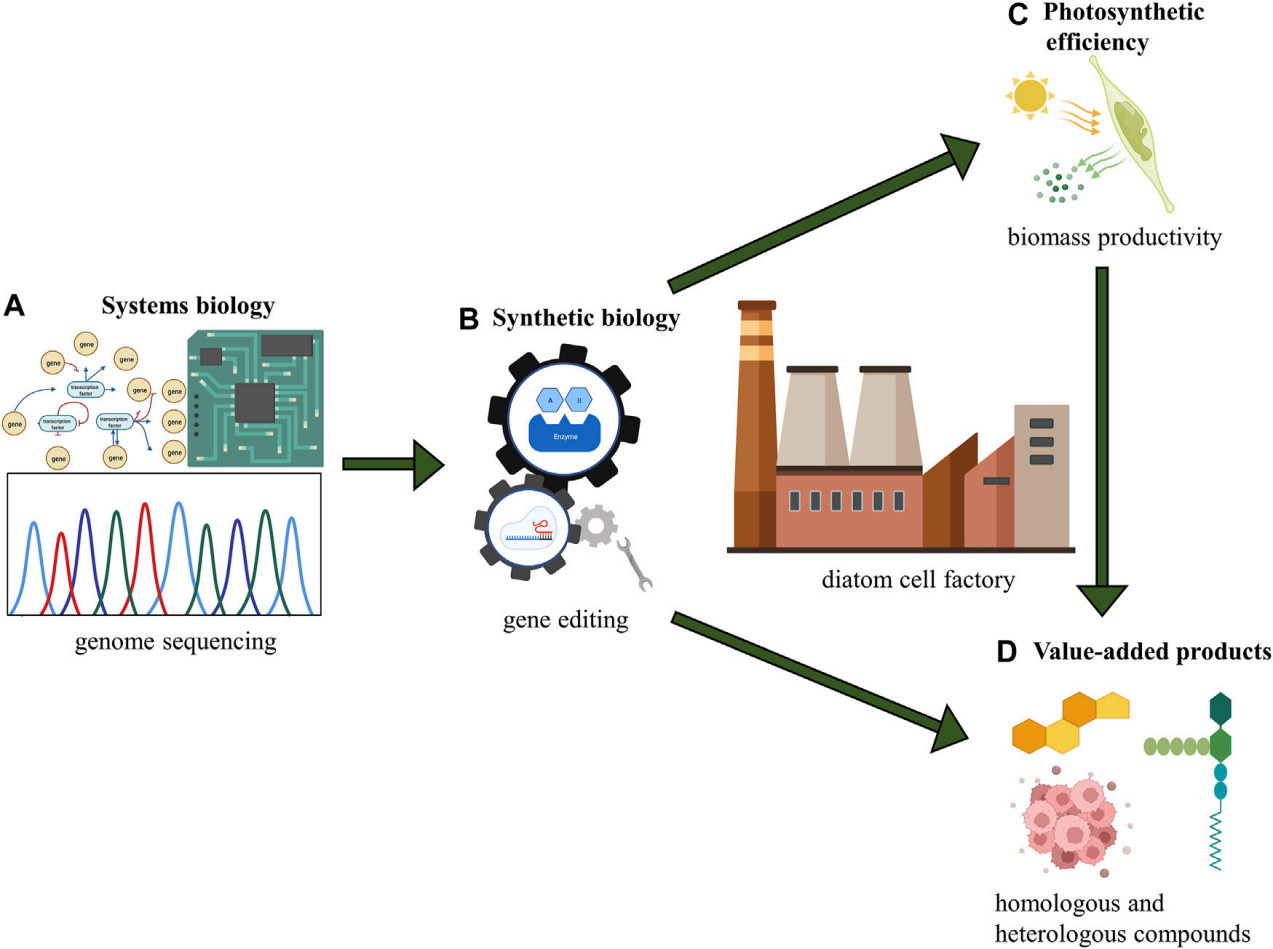
Conceptual scheme for diatom cell factory. **(A)** systems biology: understanding the whole picture of metabolism in diatoms; **(B)** synthetic biology: genetic manipulation and genome engineering; **(C)** photosynthetic efficiency: fundamental principle of energy conversion and biomass production; **(D)** value-added products: output from diatom cell factory including homologous and heterologous compounds.

This review summarizes the current systems and synthetic biology progress for diatom cell factories toward biotechnology applications (Figure 4). The systems biology approaches, including the GEMs, have facilitated metabolic prediction and rapid development of strain engineering and metabolic engineering in diatoms. Unlike GEMs of well-studied model organisms like *E. coli*, the establishment of diatom GEM is still in its early stage. The lack of high-quality and high-quantity genome annotations impedes the development of diatoms’ system biology (Vavitsas et al., 2021). PE and biomass productivity enhancements are essential foundations and boosters for cost-effective algal production. There are also considerable gaps between diatoms and some model green microalgae in the photosynthetic field, such as the modification on CCMs and Rubisco. Although more studies have to be done on diatoms, it is clear that the long-awaited breakthrough in PE will be realized under the recent progress in synthetic biology, therefore boosting biomass productivity to a brand-new level in diatoms.

Although the knowledge of the biosynthetic steps of bioactive compounds and genetic engineering help improve the yield and productivity of value-added compounds, diatom synthetic biology is still in its infancy. The essential enzymes and genes of interest, together with their regulation, are not fully understood. The diversity of the genome also poses a significant challenge to developing efficient, convenient, and stable transgenic tools for practical application since there has been no report of using the popular CRISPR technology for heterologous expression in diatoms yet. However, with the continuous development of gene-editing technology, new tools such as TALENs and CRISPR/Cas9 play a critical role in producing value-added compounds in diatoms. The combination of synthetic and systems biology will also allow us to understand better the compound conversion pathways and energy flows in diatom cell factories. These new technologies will facilitate the discovery of new compounds and the improvement of their yield, making diatoms one of the best candidates for cell factories. In the foreseeable future, we can anticipate that more compounds for bioenergy, food, feed, and pharmaceutical industries will be developed through algal cell factories, which could play an essential role in the green economy.

In short, diatom cell factories may provide solutions to global challenges like rising CO_2_ and energy crises. The diversity of diatom species offers us tremendous possibilities for digging potential value-added products. More discoveries and technological breakthroughs are needed to drive diatom research from the laboratory to commercialization. Moreover, this progress requires support from government policymakers, investors, educators, and other stakeholders. We believe a sustainable diatom cell factory is promising and achievable under continuous development of systems and synthetic biology to combine PE enhancement and compound yield improvement for biosustainability and carbon neutrality.
